# Feature Selection Combining Information Theory View and Algebraic View in the Neighborhood Decision System

**DOI:** 10.3390/e23060704

**Published:** 2021-06-02

**Authors:** Jiucheng Xu, Kanglin Qu, Meng Yuan, Jie Yang

**Affiliations:** 1 College of Computer and Information Engineering, Henan Normal University, Xinxiang 453007, China; xjc@htu.edu.cn (J.X.); y_m961123@163.com (M.Y.); yj13523769852@163.com (J.Y.); 2 Engineering Technology Research Center for Computing Intelligence and Data Mining, Xinxiang 453007, China

**Keywords:** feature selection, neighborhood rough set, non-monotonicity, algebraic view, information theory view

## Abstract

Feature selection is one of the core contents of rough set theory and application. Since the reduction ability and classification performance of many feature selection algorithms based on rough set theory and its extensions are not ideal, this paper proposes a feature selection algorithm that combines the information theory view and algebraic view in the neighborhood decision system. First, the neighborhood relationship in the neighborhood rough set model is used to retain the classification information of continuous data, to study some uncertainty measures of neighborhood information entropy. Second, to fully reflect the decision ability and classification performance of the neighborhood system, the neighborhood credibility and neighborhood coverage are defined and introduced into the neighborhood joint entropy. Third, a feature selection algorithm based on neighborhood joint entropy is designed, which improves the disadvantage that most feature selection algorithms only consider information theory definition or algebraic definition. Finally, experiments and statistical analyses on nine data sets prove that the algorithm can effectively select the optimal feature subset, and the selection result can maintain or improve the classification performance of the data set.

## 1. Introduction

Today, society has entered the era of network information, the rapid development of computer and network information technology that makes data and information in various fields increase rapidly. How to dig out potential and valuable information from the massive, disordered and strong interference data has posed an unprecedented challenge to the ability of intelligent information processing, which has produced a new field of artificial intelligence research, feature selection. Among the many methods of feature selection, rough set theory is an effective way to deal with complex systems, because it does not need to provide any prior information except for the data set [1].

Rough set theory is a theory proposed by Polish scientist Pawlak in 1982 to deal with uncertain, imprecise and fuzzy problems [1]. Its basic idea is to use equivalence relations to granulate the discrete sample space into a cluster of equivalence classes that do not intersect each other, therefore describing the knowledge and concepts in the sample space. Feature selection is one of the core contents of rough set theory and application research. Rough set theory performs information granulation on the original data set, deletes redundant conditional attributes without reducing the data classification ability, and obtains a more concise description than the original data set [2,3]. Classical rough set theory can only handle discrete data well, and cannot meet the large number of continuous and mixed data (including continuous and discrete) in practical applications [4,5,6]. Even if the discretization technology is adopted [7], the important information in the data will be lost, which will ultimately affect the selection result. For this reason, Wang et al. [8] proposed the k-nearest neighborhood rough set model. Chen et al. [9] explored the granular structure, distance and metric in the neighborhood system. Yao et al. [10] studied the relationship between the 1-step neighborhood system and rough set approximation. Based on the above research, Hu et al. [11] proposed the neighborhood rough set model and successfully applied it to the feature selection, classification and uncertainty reasoning of continuous and mixed data. As a data preprocessing method, feature selection based on the neighborhood rough set has been widely used in cancer classification [12], character recognition [13] and facial expression feature selection [14], and has good research value and application prospect.

The traditional feature selection methods have been proven to be NP hard problem by Wong and Ziarko [15]. Therefore, in the research of feature selection algorithms, how to speed up the convergence speed to reduce the time complexity has become a mainstream research direction [16]. Chen et al. [17] proposed a heuristic feature selection algorithm using joint entropy measurement. Jiang et al. [16] studied the feature selection accelerator based on the supervised neighborhood. Most of the above feature selection methods are based on monotonic evaluation functions to achieve feature selection [11]. However, the feature selection algorithm that satisfies the monotonicity has the problem that when the classification performance of the original data set is poor, the measured value of the evaluation function is low, and the final reduction effect is not good [18]. To solve this problem, Li et al. [19] proposed a non-monotonic feature selection algorithm based on decision rough set model. Sun et al. [18] designed a gene feature selection algorithm based on the uncertainty measurement of neighborhood entropy. Wang et al. [20] studied a greedy feature selection algorithm based on non-monotonic conditional discriminant index.

Some existing uncertainty measures cannot objectively reflect changes in classification decision capability [21]. Sun et al. [18] believes that credibility and coverage can reflect the classification ability of condition attributes relative to decision attributes, and condition attributes with higher credibility and coverage are more important for decision attributes. In addition, Tsumoto et al. [22] also emphasizes that credibility represents the sufficiency of propositions and coverage describes the necessity of propositions. Therefore, this paper defines the credibility and coverage in the neighborhood decision system, namely neighborhood credibility and neighborhood coverage.

The information theory definition based on information entropy and the algebraic definition based on approximate precision are two definitions form in the classic rough set theory [23]. The information theory definition based on information entropy considers the influence of attributes on uncertain subsets, while the algebraic definition based on approximate precision considers the influence of attributes on defined subsets [24,25], which are two measurement mechanisms with strong complementarity [26]. So far, most feature selection algorithms only consider information theory definition or algebraic definition. For example, Hu et al. [11] proposed a hybrid feature selection algorithm based on neighborhood information entropy. Wang et al. [27,28] used the equivalent relation matrix to calculate the concepts of knowledge granularity, resolution and attribute importance from the algebraic view of rough sets. Sun et al. [2,29] studied the feature selection method based on entropy measures. The uncertainty measures based on neighborhood information entropy reflect the information theory view in the neighborhood decision system, and the neighborhood approximate precision belongs to the algebraic view in the neighborhood decision system [18].

Inspired by the above, this paper combines the information theory view and algebra view in the neighborhood decision system, and proposes a heuristic non-monotonic feature selection algorithm. The experimental results on nine different scale data sets show that the algorithm can effectively select the optimal feature subset, and the selection results can maintain or improve the classification performance of the data set.

In summary, the main contributions of this paper are as follows:

- The credibility and coverage degrees can reflect the decision-making ability and the classification ability of conditional attributes with respect to the decision attribute [18]. In order to effectively analyze the uncertainty of knowledge in the neighborhood rough set, the credibility and coverage are introduced into the neighborhood decision system, and then the neighborhood credibility and neighborhood coverage are defined and introduced into neighborhood joint entropy.
- Based on the proposed neighborhood joint entropy, some uncertainty measures of neighborhood information entropy are studied, and the relationship between the measures is derived, which is conducive to understanding the nature of knowledge uncertainty in neighborhood decision systems.
- To construct a more comprehensive measurement mechanism and overcome the problem of poor selection results when the classification performance of the original data set is not good, the information theory view and algebraic view in the neighborhood decision system are combined to propose a heuristic non-monotonic feature selection algorithm.

Section 2 briefly introduces the basic concepts of the neighborhood rough set and information entropy measures. Section 3 studies the heuristic non-monotonic feature selection algorithm based on information theory view and algebraic view. Section 4 analyzes the experimental results on four low-dimensional data sets and five high-dimensional data sets. Section 5 summarizes the content of this paper.

## 2. Basic Concepts

In this part, we will briefly review the basic concepts of information entropy measures and the neighborhood rough set [2,30,31,32,33].

### 2.1. Information Entropy Measures

$DS=\left(U,C\cup D,\;V,f\right)$ is called a decision system, where $U=\left\{x_{1},x_{2},\ldots ,x_{k}\right\}$ is the sample set, *C* is the conditional attribute set, *D* is the classification decision attribute, *V* is the value of attribute, f:U×C→V is a mapping function.

In the DS, if B⊆C divides the sample set *U* into $U/B=\left\{X_{1},X_{2},\ldots ,X_{K}\right\}$, then the information entropy is defined as

(1) $$H\left(B\right)=-\sum_{i=1}^{K}p\left(X_{i}\right)\log p\left(X_{i}\right)\;\;\;\;X_{i}\subseteq U/B$$

$p\left(X_{i}\right)=\frac{\left|X_{i}\right|}{\left|U\right|}$ represents the probability of $X_{i}$ in the sample set.

In the DS, if B,Q⊆C, $U/B=\left\{X_{1},X_{2},\ldots ,X_{K}\right\}$, $U/Q=\left\{Y_{1},Y_{2},\ldots ,Y_{L}\right\}$, then the conditional information entropy of *Q* relative to *B* is defined as

(2) $$H\left(Q|B\right)=-\sum_{i=1}^{K}p\left(X_{i}\right)\sum_{j=1}^{L}p\left(Y_{j}|X_{i}\right)\log p\left(Y_{j}|X_{i}\right)\;\;$$

where $X_{i}\subseteq U/B,\;Y_{j}\subseteq U/Q,\;p\left(Y_{j}|X_{i}\right)=\frac{\left|Y_{j}\cap X_{i}\right|}{|X_{i}|}$.

In the DS, if B,Q⊆C, $U/B=\left\{X_{1},X_{2},\ldots ,X_{K}\right\}$, $U/Q=\left\{Y_{1},Y_{2},\ldots ,Y_{L}\right\}$, then the joint information entropy of *Q* and *B* is defined as

(3) $$H\left(Q,B\right)=-\sum_{i=1}^{K}\sum_{j=1}^{L}p\left(X_{i}\cap Y_{j}\right)log\left(p\left(X_{i}\cap Y_{j}\right)\right)$$

where $X_{i}\subseteq U/B,\;Y_{j}\subseteq U/Q,p\left(X_{i}\cap Y_{j}\right)=\frac{\left|X_{i}\cap Y_{j}\right|}{\left|U\right|}$.

**Theorem** **1.** *Given the DS, if B,Q⊆C, $U/B=\left\{X_{1},X_{2},\ldots ,X_{K}\right\}$, $U/Q=\left\{Y_{1},Y_{2},\ldots ,Y_{L}\right\}$, then $H\left(Q|B\right)\;=H\left(Q,B\right)-H\left(B\right)$.*

### 2.2. Neighborhood Rough Set

$NDS=\left(U,C,D,\;\delta \right)$ is called the neighborhood decision system, where *U* is a sample set named universe, *C* is the conditional attribute set, *D* is decision attribute, and δ is the neighborhood radius.

In the NDS, if B⊆C, then Minkowski distance between different sample points $x_{i}=\left\{x_{i1},x_{i2},\ldots ,x_{im}\right\}$ and $x_{j}=\left\{x_{j1},x_{j2},\ldots ,x_{jm}\right\}$ on *U* is defined as

(4) MDBxi, xj= ∑k=1Bxik−xjkp11pp

Given the NDS and the distance measurement function MD, if B⊆C, then the neighborhood information granule of $x_{i}\in U$ relative to *B* is defined as

(5) $$n_{B}^{\delta }\left(x_{i}\right)=\left\{x\in U|\Delta_{B}\left(x_{i}\;,\;x\right)\leq \delta \right\}\;\;\;\;\;\;\;\;\delta >0$$

$n_{B}^{\delta }\left(x_{i}\right)$ represents the indistinguishable relation sample set of the $x_{i}$ under *B*.

In the NDS, if $U/D=\{Y_{1},Y_{2},\ldots ,Y_{L}\}$, then the decision equivalence relation of $x_{i}\in U$ is defined as

(6) $${\left[x_{i}\right]}_{D}=\left\{Y_{j}|x_{i}\in Y_{j}\right\}\;\;\;\;\;j=1,2\ldots ,L$$

In the NDS, if B⊆C, $N_{B}$ is the neighborhood relationship on *U*, then the neighborhood upper approximation set $\bar{N_{B}}X$ and the neighborhood lower approximation set $\underline{N_{B}}X$ of sample set X ⊆ U relative to *B* are respectively defined as

(7) $$\bar{N_{B}}X=\left\{x_{i}\in U|n_{B}^{\delta }\left(x_{i}\right)\cap X\;\neq \emptyset \right\}\;\;i=1,2\ldots ,\left|U\right|$$

(8) $$\underline{N_{B}}X=\left\{x_{i}\in U|n_{B}^{\delta }\left(x_{i}\right)\subseteq X\right\}\;\;\;i=1,2\ldots ,\left|U\right|$$

In the NDS, if B⊆C, $U/D=\{Y_{1},Y_{2},\ldots ,Y_{L}\}$, $N_{B}$ is the neighborhood relationship on *U*, then the upper approximate set $\bar{N_{B}}\left(D\right)$ and the lower neighborhood approximate set $\underline{N_{B}}\left(D\right)$ of *D* relative to *B* are respectively defined as

(9) $$\bar{N_{B}}\left(D\right)={⋃}_{S=1}^{L}\bar{N_{B}}Y_{s}$$

(10) $$\underline{N_{B}}\left(D\right)=\;{⋃}_{S=1}^{L}\underline{N_{B}}Y_{s}$$

In the NDS, if B⊆C, then the neighborhood approximate precision of the sample set X ⊆ U relative to *B* is defined as

(11) $$P_{B}\left(X\right)=\;\frac{\left|\underline{N_{B}}\left(X\right)\right|}{\left|\bar{N_{B}}\left(X\right)\right|}$$

In the NDS, if B⊆C, $U/D=\{Y_{1},Y_{2},\ldots ,Y_{L}\}$, then the neighborhood approximate precision of *D* relative to *B* is defined as

(12) $$P_{B}\left(D\right)=\;\frac{\left|\underline{N_{B}}\left(D\right)\right|}{\left|\bar{N_{B}}\left(D\right)\right|}$$

$P_{B}\left(D\right)$ describes the knowledge completeness of a set, considering the influence of attributes in the neighborhood decision system on the defined subset, and is the view of the neighborhood decision system under algebraic definition [18].

## 3. Feature Selection Algorithm Design

This part first defines the neighborhood credibility and neighborhood coverage. Second, some uncertainty measures of neighborhood information entropy are studied, and the relationship between the measures is derived. Then, using the information theory view and algebraic view in the neighborhood decision system, a heuristic non-monotonic feature selection algorithm is designed. The following introduces related concepts and their properties.

### 3.1. Neighborhood Credibility and Neighborhood Coverage

In the NDS, if B ⊆ C, $U/B=\left\{X_{1},X_{2},\ldots ,X_{K}\right\}$, $U/D=\left\{Y_{1},Y_{2},\ldots ,Y_{L}\right\}$, then the credibility $\alpha_{ij}$ and coverage $\kappa_{ij}$ [18] are respectively defined as

(13) $$\alpha_{ij}=\frac{\left|X_{i}\cap Y_{j}\right|}{\left|X_{i}\right|}$$

(14) $$\kappa_{ij}=\frac{\left|X_{i}\cap Y_{j}\right|}{\left|Y_{j}\right|}$$

where i=1,2,…,K   and j=1,2,…,L. Credibility and coverage reflect the classification ability of condition attributes relative to decision attributes. Condition attributes with higher credibility and coverage are more important for decision attributes [22].

**Definition** **1.** *In the*NDS, if B⊆C, then the joint neighborhood information granule of $x_{i}\in U$*is defined as*

(15) $$n_{\left(B,D\right)}\left(x_{i}\right)=n_{B}^{\delta }\left(x_{i}\right)\cup {\left[x_{i}\right]}_{D}$$

*$n_{\left(B,D\right)}\left(x_{i}\right)$ combines the neighborhood information granule $n_{B}^{\delta }\left(x_{i}\right)$ and decision equivalence relationship ${\left[x_{i}\right]}_{D}$, which more accurately reflects the amount of class information when each class in $n_{B}^{\delta }\left(x_{i}\right)$ has a different distribution, and the amount of class information provided is embodied in the number of elements in $n_{\left(B,D\right)}\left(x_{i}\right)$. Therefore, $n_{\left(B,D\right)}\left(x_{i}\right)$ can accurately reflect the decision information.*

**Definition** **2.** *In the NDS, if B⊆C, then the neighborhood credibility $n\alpha_{i}$ and neighborhood coverage $n\kappa_{i}$ of $x_{i}\in U$ are respectively defined as*

(16) $$n\alpha_{i}=\frac{\left|n_{B}^{\delta }\left(x_{i}\right)\cap {\left[x_{i}\right]}_{D}\right|}{|n_{\left(B,D\right)}\left(x_{i}\right))|}$$

(17) $$n\kappa_{i}=\frac{\left|n_{B}^{\delta }\left(x_{i}\right)\cap {\left[x_{i}\right]}_{D}\right|}{\left|{\left[x_{i}\right]}_{D}\right|}$$

*$n\alpha_{i}$ and $n\kappa_{i}$ respectively use the joint neighborhood information granule and the decision equivalence relationship to describe the credibility and coverage of the neighborhood decision system, which makes full use of the decision information provided by the decision system.*

### 3.2. Uncertainty Measures of Neighborhood Information Entropy

In the NDS, if B⊆C, then neighborhood entropy [34] of $x_{i}\in U$ is defined as

(18) $$H_{\delta }^{x_{i}}\left(B\right)=\;-\log\left(\frac{\left|n_{B}^{\delta }\left(x_{i}\right)\right|}{\left|U\right|}\right)$$

In the NDS, if B⊆C, then the average neighborhood entropy [34] is defined as

(19) $$H_{\delta }\left(B\right)=\;\frac{1}{\left|U\right|}\sum_{i=1}^{\left|U\right|}H_{\delta }^{x_{i}}\left(B\right)=-\frac{1}{\left|U\right|}\sum_{i=1}^{\left|U\right|}\log\left(\frac{\left|n_{B}^{\delta }\left(x_{i}\right)\right|}{\left|U\right|}\right)$$

**Definition** **3.** *In the NDS, if B⊆C, then new neighborhood entropy of $x_{i}\in U$ is defined as*

(20) $$H_{\delta }^{x_{i}}\left(B\right)=\;-\log\left(\frac{\left|n_{B}^{\delta }\left(x_{i}\right)\right|}{|n_{\left(B,D\right)}\left(x_{i}\right)}\right)$$

**Definition** **4.** *In the NDS, if B⊆C, then the new average neighborhood entropy is defined as*

(21) $$H_{\delta }\left(B\right)=\frac{P_{B}\left(D\right)}{\left|U\right|}\sum_{i=1}^{\left|U\right|}H_{\delta }^{x_{i}}\left(B\right)=-\frac{P_{B}\left(D\right)}{\left|U\right|}\sum_{i=1}^{\left|U\right|}\log\left(\frac{\left|n_{B}^{\delta }\left(x_{i}\right)\right|}{|n_{\left(B,D\right)}\left(x_{i}\right)|}\right)$$

*The new average neighborhood entropy $H_{\delta }\left(B\right)$ introduces the joint neighborhood information granule into neighborhood entropy, which makes full use of the decision information in the neighborhood decision system.*

**Definition** **5.** *In the NDS, if B⊆C, then neighborhood conditional entropy of D relative to B is defined as*

(22) $$H_{\delta }\left(D|B\right)=\;-\frac{P_{B}\left(D\right)}{\left|U\right|}\sum_{i=1}^{\left|U\right|}\log\left(\frac{{\left|n_{B}^{\delta }\left(x_{i}\right)\cap {\left[x_{i}\right]}_{D}\right|}^{2}}{\left|n_{B}^{\delta }\left(x_{i}\right)\right|\left|{\left[x_{i}\right]}_{D}\right|}\right)$$

**Definition** **6.** *In the NDS, if B⊆C, then neighborhood joint entropy of D and B is defined as*

(23) $$H_{\delta }\left(D,B\right)=-\frac{P_{B}\left(D\right)}{\left|U\right|}\sum_{i=1}^{\left|U\right|}\log\left(\frac{{\left|n_{B}^{\delta }\left(x_{i}\right)\cap {\left[x_{i}\right]}_{D}\right|}^{2}}{\left|n_{\left(B,D\right)}\left(x_{i}\right)\right|\left|{\left[x_{i}\right]}_{D}\right|}\right)\;\;$$

**Theorem** **2.** Given the NDS, if B⊆C, then $H_{\delta }\left(D,B\right)=-\frac{P_{B}\left(D\right)}{\left|U\right|}\sum_{i=1}^{\left|U\right|}\log\left(n\kappa_{i}*n\alpha_{i}\right)$.

**Proof** **of** **Theorem 2** $$\begin{array}{cc} \;H_{\delta }\left(D,B\right) & =-\frac{P_{B}\left(D\right)}{\left|U\right|}\sum_{i=1}^{\left|U\right|}\log\left(\frac{{\left|n_{B}^{\delta }\left(x_{i}\right)\cap {\left[x_{i}\right]}_{D}\right|}^{2}}{\left|n_{\left(B,D\right)}\left(x_{i}\right)\right|\left|{\left[x_{i}\right]}_{D}\right|}\right)\\ \; & =-\frac{P_{B}\left(D\right)}{\left|U\right|}\sum_{i=1}^{\left|U\right|}\log\left(\frac{\left|n_{B}^{\delta }\left(x_{i}\right)\cap {\left[x_{i}\right]}_{D}\right|\left|n_{B}^{\delta }\left(x_{i}\right)\cap {\left[x_{i}\right]}_{D}\right|}{\left|n_{\left(B,D\right)}\left(x_{i}\right)\right|\left|{\left[x_{i}\right]}_{D}\right|}\right)\\ \; & =-\frac{P_{B}\left(D\right)}{\left|U\right|}\sum_{i=1}^{\left|U\right|}\log\left(\frac{\left|n_{B}^{\delta }\left(x_{i}\right)\cap {\left[x_{i}\right]}_{D}\right|}{\left|n_{\left(B,D\right)}\left(x_{i}\right)\right|}\frac{\left|n_{B}^{\delta }\left(x_{i}\right)\cap {\left[x_{i}\right]}_{D}\right|}{\left|{\left[x_{i}\right]}_{D}\right|}\right)\\ \; & =-\frac{P_{B}\left(D\right)}{\left|U\right|}\sum_{i=1}^{\left|U\right|}\log\left(n\alpha_{i}*\;n\kappa_{i}\right) \end{array}$$

From Theorem 2, we can see that the definition of neighborhood joint entropy can be derived from neighborhood credibility and neighborhood coverage.   □

**Theorem** **3.** *Given the NDS, if B⊆C, then $H_{\delta }\left(D|B\right)=H_{\delta }\left(D,B\right)-H_{\delta }\left(B\right)$.*

**Proof** **of** **Theorem 3** $$\begin{array}{cc} H_{\delta }\left(D,B\right)-H_{\delta }\left(B\right) & =-\frac{P_{B}\left(D\right)}{\left|U\right|}\sum_{i=1}^{\left|U\right|}\log\left(\frac{{\left|n_{B}^{\delta }\left(x_{i}\right)\cap {\left[x_{i}\right]}_{D}\right|}^{2}}{\left|n_{\left(B,D\right)}\left(x_{i}\right)\right|\left|{\left[x_{i}\right]}_{D}\right|}\right)+\frac{P_{B}\left(D\right)}{\left|U\right|}\sum_{i=1}^{\left|U\right|}\log\left(\frac{\left|n_{B}^{\delta }\left(x_{i}\right)\right|}{\left|n_{\left(B,D\right)}\left(x_{i}\right)\right|}\right)\\ \; & =-\frac{P_{B}\left(D\right)}{\left|U\right|}\sum_{i=1}^{\left|U\right|}\log\left(\frac{{\left|n_{B}^{\delta }\left(x_{i}\right)\cap {\left[x_{i}\right]}_{D}\right|}^{2}}{\left|n_{\left(B,D\right)}\left(x_{i}\right)\right|\left|{\left[x_{i}\right]}_{D}\right|}\right)+\frac{P_{B}\left(D\right)}{\left|U\right|}\sum_{i=1}^{\left|U\right|}\log\left(\frac{\left|n_{B}^{\delta }\left(x_{i}\right)\right|}{\left|n_{\left(B,D\right)}\left(x_{i}\right)\right|}\right)\\ \; & =-\frac{P_{B}\left(D\right)}{\left|U\right|}\sum_{i=1}^{\left|U\right|}\log\left(\frac{{\left|n_{B}^{\delta }\left(x_{i}\right)\cap {\left[x_{i}\right]}_{D}\right|}^{2}}{\left|n_{\left(B,D\right)}\left(x_{i}\right)\right|\left|{\left[x_{i}\right]}_{D}\right|}\frac{\left|n_{\left(B,D\right)}\left(x_{i}\right)\right|}{\left|n_{B}^{\delta }\left(x_{i}\right)\right|}\right)\\ \; & =-\frac{P_{B}\left(D\right)}{\left|U\right|}\sum_{i=1}^{\left|U\right|}\log\left(\frac{{\left|n_{B}^{\delta }\left(x_{i}\right)\cap {\left[x_{i}\right]}_{D}\right|}^{2}}{\left|n_{B}^{\delta }\left(x_{i}\right)\right|\left|{\left[x_{i}\right]}_{D}\right|}\right) \end{array}$$

According to *Definition 5*, $H_{\delta }\left(D|B\right)=H_{\delta }\left(D,B\right)-H_{\delta }\left(B\right)$ holds.    □

Sun et al. [18] shows that information entropy and its extension belong to the view under the information theory definition, and the neighborhood approximate precision comes from the view under the algebra definition. Therefore, Definitions 4–6 can be used to measure the uncertainty of knowledge in the neighborhood decision system from the information theory view and the algebraic view.

### 3.3. Heuristic Non-Monotonic Feature Selection Algorithm Design

The feature selection algorithm that satisfies the monotonicity has the problem that the reduction effect is not good when the classification performance of the original data set is poor. Therefore, based on the uncertainty measures combining algebraic view and information theory view in Section 3.2, a heuristic non-monotonic feature selection algorithm is designed.

**Theorem** **4.** *Given the NDS, if $B_{1}\subseteq B_{2}\subseteq C$, then $H_{\delta }\left(D,B\right)$ is non-monotonic.*

**Proof** **of** **Theorem 4.** we can know that $\left|n_{B_{1}}^{\delta }\left(x_{i}\right)\right|\geq \left|n_{B_{2}}^{\delta }\left(x_{i}\right)\right|$, so $\left|n_{B_{1}}^{\delta }\left(x_{i}\right)\cap {\left[x_{i}\right]}_{D}\right|\geq$$\left|n_{B_{2}}^{\delta }\left(x_{i}\right)\cap {\left[x_{i}\right]}_{D}\right|$, $\left|n_{B_{1}}^{\delta }\left(x_{i}\right)\cup {\left[x_{i}\right]}_{D}\right|\geq \left|n_{B_{2}}^{\delta }\left(x_{i}\right)\cup {\left[x_{i}\right]}_{D}\right|$ and $\;\left|n_{\left(B_{1},D\right)}\left(x_{i}\right)\right|\geq \left|n_{\left(B_{2},D\right)}\left(x_{i}\right)\right|$ from Equation (5). Then it can be deduced that the numerical relationship between $\frac{{\left|n_{B_{1}}^{\delta }\left(x_{i}\right)\cap {\left[x_{i}\right]}_{D}\right|}^{2}}{\left|n_{\left(B_{1},D\right)}\left(x_{i}\right)\right|}$ and $\frac{{\left|n_{B_{2}}^{\delta }\left(x_{i}\right)\cap {\left[x_{i}\right]}_{D}\right|}^{2}}{\left|n_{\left(B_{2},D\right)}\left(x_{i}\right)\right|}$ is not clear, so the numerical relationship between $-\frac{1}{\left|U\right|}\sum_{i=1}^{\left|U\right|}\log\left(\frac{{\left|n_{B_{1}}^{\delta }\left(x_{i}\right)\cap {\left[x_{i}\right]}_{D}\right|}^{2}}{\left|n_{\left(B_{1},D\right)}\left(x_{i}\right)\right|\left|{\left[x_{i}\right]}_{D}\right|}\right)$ and $-\frac{1}{\left|U\right|}\sum_{i=1}^{\left|U\right|}\log\left(\frac{{\left|n_{B_{2}}^{\delta }\left(x_{i}\right)\cap {\left[x_{i}\right]}_{D}\right|}^{2}}{\left|n_{\left(B_{2},D\right)}\left(x_{i}\right)\right|\left|{\left[x_{i}\right]}_{D}\right|}\right)$ is unknown. According to Equations (9), (10) and (12), we can obtain $P_{B_{1}}\left(D\right)\leq P_{B_{2}}\left(D\right)$, so value relationship of $-\frac{P_{B_{1}}\left(D\right)}{\left|U\right|}\sum_{i=1}^{\left|U\right|}\log\left(\frac{{\left|n_{B_{1}}^{\delta }\left(x_{i}\right)\cap {\left[x_{i}\right]}_{D}\right|}^{2}}{\left|n_{\left(B_{1},D\right)}\left(x_{i}\right)\right|\left|{\left[x_{i}\right]}_{D}\right|}\right)$ and $-\frac{P_{B_{2}}\left(D\right)}{\left|U\right|}\sum_{i=1}^{\left|U\right|}\log\left(\frac{{\left|n_{B_{2}}^{\delta }\left(x_{i}\right)\cap {\left[x_{i}\right]}_{D}\right|}^{2}}{\left|n_{\left(B_{2},D\right)}\left(x_{i}\right)\right|\left|{\left[x_{i}\right]}_{D}\right|}\right)$ are uncertain. According to Equation (23), Theorem 4 holds.    □

**Definition** **7.** *In the NDS, if B⊆C, attribute b∈B  satisfies $H_{\delta }\left(D,B\right)\leq H_{\delta }\left(D,B-\left\{b\right\}\right)$, then it is said that attribute b is redundant with respect to D, otherwise it is said that attribute b is indispensable for D. If B satisfies the following conditions, then B is called a feature subset of C.*

*(1) $H_{\delta }\left(D,B\right)\geq \;H_{\delta }\left(D,C\right)$*

*(2) $H_{\delta }\left(D,B\right)>H_{\delta }\left(D,\left\{B-b\right\}\right)\;\;\;\;\;\;\;\forall b\in B$*

**Definition** **8.** *In the NDS, if B⊆C, then the importance of attribute $b\in \left(C-B\right)$ is defined as*

(24) $$Sig\left(b,B,D\right)=H_{\delta }\left(D,B\cup \left\{b\right\}\right)-H_{\delta }\left(D,B\right)$$

*when B= ∅, $Sig\left(b,B,D\right)=H_{\delta }\left(D,\left\{b\right\}\right)$. The larger $Sig\left(b,B,D\right)$, the more important b is. From a numerical point of view, looking for an optimal feature subset is to find the B corresponding to the maximum $H_{\delta }\left(D,B\right)$.*

To accurately reflect the decision information and eliminate redundant features, a heuristic non-monotonic feature selection algorithm based on neighborhood joint entropy (BONJE) is designed. The implementation steps of this algorithm are shown in Algorithm 1.
**Algorithm 1:** B0NJE Algorithm Steps.**Input**: Given the NDS**Output**: A feature subset *B*1. Initialize B=Agent=∅, $H_{\delta }\left(D,B\right)=0$2. **While** $Sig\left(C,B,D\right)\leq 0$ **do**3.  Let H=04.  **for** any $b\in \left(C-B\right)$ **do**5.    Calculate $H_{\delta }\left(D,B\cup b\right)$6.    **if**$H_{\delta }\left(D,B\cup b\right)\;>H$ **then**7.      Let Agent=B∪b and $H=H_{\delta }\left(D,B\cup b\right)$8.    **end if**9.  **end for**10.  Let B=Agent11. **end while**12. **return** A feature subset *B*


To facilitate the understanding of the specific calculation steps of the algorithm, an example is given below.

**Example** **1.** *A $NDS=\left(U,C,D,\;\delta \right)$ is given in Table 1, where $\;U=\left\{x_{1},x_{2},x_{3},x_{4}\right\}$ is the universe, $C=\left\{a,b,c\right\}$ is the conditional attribute set, D=d is the decision attribute, and the neighborhood radius parameter δ=0.3.*

Let the initial feature subset B=∅, the base of log is 10, the calculation result is kept to three decimal places. In the distance measurement function Equation (Section 2.2), p=2 is used as the calculation function.

From Equation (6), we know that ${\left[x_{1}\right]}_{D}=\left\{x_{1},x_{2}\right\}$, ${\left[x_{2}\right]}_{D}=\left\{x_{1},x_{2}\right\}$, ${\left[x_{3}\right]}_{D}=\left\{x_{3},x_{4}\right\}$, ${\left[x_{4}\right]}_{D}=\left\{x_{3},x_{4}\right\}$.

When B = a, the distance between each sample is as follows: $MD_{\left\{a\right\}}\left(x_{1},x_{1}\right)=0\leq \delta$, $MD_{\left\{a\right\}}\left(x_{1},x_{2}\right)=0.09\leq \delta$, $MD_{\left\{a\right\}}\left(x_{1},x_{3}\right)=0.19\leq \delta$, $MD_{\left\{a\right\}}\left(x_{1},x_{4}\right)=0.49\geq \delta$, $MD_{\left\{a\right\}}\left(x_{2},x_{3}\right)=0.1\leq \delta$, $MD_{\left\{a\right\}}\left(x_{2},x_{4}\right)=0.4\geq \delta$, $MD_{\left\{a\right\}}\left(x_{3},x_{4}\right)=0.3\leq \delta$.

According to Equation (5), we obtain $n_{\left\{a\right\}}^{\delta }\left(x_{1}\right)=\left\{x_{1},x_{2},x_{3}\right\},n_{\left\{a\right\}}^{\delta }\left(x_{2}\right)=\left\{x_{1},x_{2},x_{3}\right\}$, $n_{\left\{a\right\}}^{\delta }\left(x_{3}\right)=\left\{x_{1},x_{2},x_{3},x_{4}\right\}$, $n_{\left\{a\right\}}^{\delta }\left(x_{4}\right)=\left\{x_{3},x_{4}\right\}$.

We know that $\;n_{\left(\left\{a\right\},D\right)}\left(x_{1}\right)=n_{\left\{a\right\}}\left(x_{1}\right)\cup {\left[x_{1}\right]}_{D}=\left\{x_{1},x_{2},x_{3}\right\}$, $n_{\left(\left\{a\right\},D\right)}\left(x_{2}\right)=n_{\left\{a\right\}}\left(x_{2}\right)$$\cup {\left[x_{2}\right]}_{D}=\left\{x_{1},x_{2},x_{3}\right\}$, $n_{\left(\left\{a\right\},D\right)}\left(x_{3}\right)=n_{\left\{a\right\}}\left(x_{3}\right)\cup {\left[x_{3}\right]}_{D}=\left\{x_{1},x_{2},x_{3},x_{4}\right\}$, $n_{\left(\left\{a\right\},D\right)}\left(x_{4}\right)=n_{\left\{a\right\}}\left(x_{4}\right)\cup {\left[x_{4}\right]}_{D}=\left\{x_{3},x_{4}\right\}$ from *Equation* (15).

From Equations (9), (10) and (12), we can obtain $\bar{N_{\left\{a\right\}}}\left(D\right)=\left\{x_{1},x_{2},x_{3},x_{4}\right\}$, $\underline{N_{\left\{a\right\}}}\left(D\right)=\left\{x_{4}\right\}$, $P_{\left\{a\right\}}\left(X\right)=\frac{\left|\underline{N_{\left\{a\right\}}}\left(D\right)\right|}{\left|\bar{N_{\left\{a\right\}}}\left(D\right)\right|}=\frac{1}{4}$ respectively.

According to Equation (23), we can obtain $H_{\delta }\left(D,\left\{a\right\}\right)=-\frac{P_{B}\left(D\right)}{\left|U\right|}\sum_{i=1}^{\left|U\right|}\log\left(\frac{{\left|n_{B}^{\delta }\left(x_{i}\right)\cap {\left[x_{i}\right]}_{D}\right|}^{2}}{\left|n_{\left(B,D\right)}\left(x_{i}\right)\right|\left|{\left[x_{i}\right]}_{D}\right|}\right)$=−1144( ( 223 2 ) + ( 223 2 ) + ( 224 2 ) + ( 222 2 ) ) = 0.041

Similarly, $H_{\delta }\left(D,\left\{b\right\}\right)=0$, $H_{\delta }\left(D,\left\{c\right\}\right)=0.116$, $H_{\delta }\left(D,\left\{a,b\right\}\right)=0.195$, $H_{\delta }\left(D,\left\{a,c\right\}\right)=0.345$, $H_{\delta }\left(D,\left\{b,c\right\}\right)=0.116$, $H_{\delta }\left(D,\left\{a,b,c\right\}\right)=0.345$.

It can be seen from the results that $H_{\delta }\left(D,\left\{b\right\}\right)<H_{\delta }\left(D,\left\{a\right\}\right)<H_{\delta }\left(D,\left\{c\right\}\right)$, so add $\left\{c\right\}$ to *B*. Since $H_{\delta }\left(D,\left\{c\right\}\right)=H_{\delta }\left(D,\left\{b,c\right\}\right)<H_{\delta }\left(D,\left\{a,c\right\}\right)$, so add $\left\{a\right\}$ to *B*. $H_{\delta }\left(D,\left\{a,b,c\right\}\right)=H_{\delta }\left(D,\left\{a,c\right\}\right)$ meets the suspension requirement, so $B=\left\{a,c\right\}$ is the optimal feature subset.

## 4. Experiment and Analysis

This part uses the BONJE algorithm to select the appropriate neighborhood radius for different data sets and designs different comparative experiments to prove the efficiency of the BONJE algorithm in feature selection.

### 4.1. Experimental Data Introduction

To verify the efficiency of the BONJE algorithm in feature selection, this experiment selects nine data sets with different dimensions as the experimental objects, including 4 low-dimensional data sets (Wine, WDBC, WPBC, Ionosphere) and 5 high-dimensional data sets (Colon, SRBCT, DLBCL, Leukemia, Lung). The specific data of each data set is shown in Table 2.

Wine, WDBC (Wisconsin Diagnostic Breast Cancer), WPBC (Wisconsin Prognostic Breast Cancer), Ionosphere data sets are downloaded at https://archive.ics.uci.edu/ml/datasets.html (accessed on 31 May 2021). Colon data set is downloaded from http://eps.upo.es/bigs/datasets.html (accessed on 31 May 2021). SRBCT (Small Round Blue Cell Tumor) data set. DLBCL (Diffuse Large B Cell Lymphoma), Leukemia data sets are downloaded from http://www.gems-system.org. (accessed on 31 May 2021). Lung data set is downloaded from http://bioinformatics.rutgers.ed/Static/Supplemens/CompCancer/datasets (accessed on 31 May 2021).

### 4.2. Experimental Environment

The experiment in this paper is performed on a personal computer with Microsoft Windows 10 Professional Edition (64-bit), (Intel) Intel(R) Core(TM) i5-6500 CPU @ 3.20 GHz (3192 MHz) and 16.00 GB RAM. The simulation experiment is implemented on the IntelliJ IDEA 2020.1.2 platform using Java version “1.8.0_144”. C4.5, SVM (support vector machine) and KNN (k-nearest neighbors) classifiers are selected on Weka software to verify the classification accuracy of selected feature subsets, where SVM uses PolyKernel as the kernel function, and KNN sets K = 3. In order to reduce the generalization error, the three classifiers all adopt a ten-fold cross-validation method to obtain the final classification accuracy.

### 4.3. Neighborhood Radius Selection

**Figure 1:**
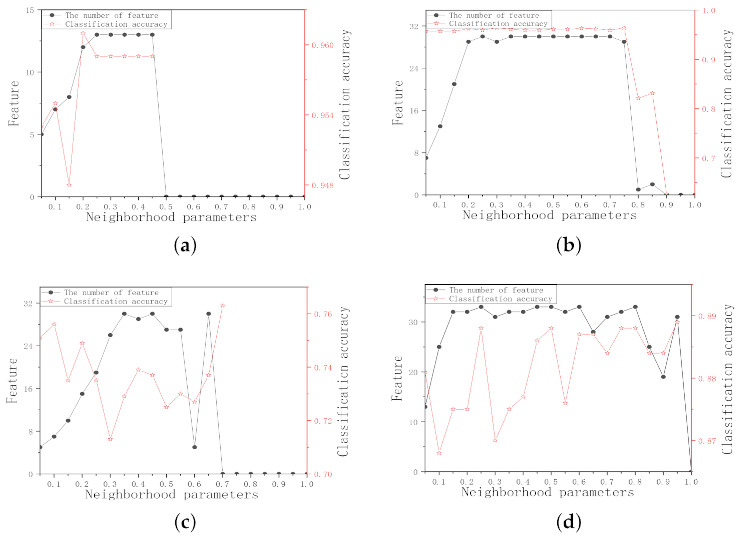

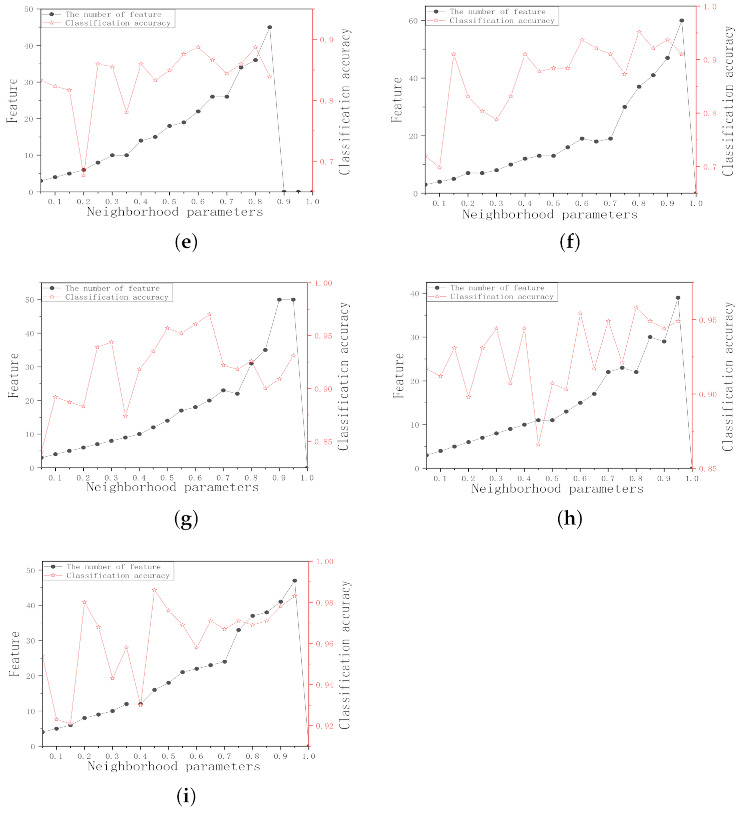
The number of selected features and average classification accuracy of nine data sets in different neighborhood radii. (**a**) Wine. (**b**) WDBC. (**c**) WPBC. (**d**) Ionosphere. (**e**) Colon. (**f**) SRBCT. (**g**) DLBCL. (**h**) Leukemia1. (**i**) Lung.

Since the neighborhood radius affects the granularity of neighborhood information, and thus neighborhood joint entropy, it is very important to choose a proper neighborhood radius. In order to unify the value of the neighborhood radius, eliminate the difference in dimensions and make each feature be treated equally by the classifier, this experiment, first, normalizes the data ($\frac{x-Min}{Max-Min}$), then the neighborhood radius is set in [0.05, 1] with 0.05 as the interval. The number of selected features and the three classifiers average classification accuracy in the different neighborhood radii are shown in Figure 1.

For Wine data set in Figure 1a, as the neighborhood radius value increases, the number of selected features increases sharply. The number of selected features is small when the neighborhood radius value is in the interval [0.05, 0.15] and the average classification accuracy reaches the highest when δ = 0.1 in this interval. Similar to Wine data set, the δ values of WDBC and WPBC data sets are set to 0.05 and 0.1, respectively. For Ionosphere data set in Figure 1d, the average classification accuracy is higher when the neighborhood radius value is in the interval [0.05, 0.2] and the number of selected features is the least when δ = 0.05 in this interval. For Colon data set in Figure 1e, the change trend of the average classification accuracy is obvious. The number of selected features is small, and the classification accuracy is higher when δ = 0.25. Similar to Colon data set, the δ values of SRBCT, DLBCL, Leukemia, and Lung data sets can be set to 0.15, 0.3, 0.3, and 0.45, respectively. Therefore, the neighborhood radius values of the 9 data sets should be within [0.05, 0.45].

### 4.4. Classification Results of Bonje Algorithm

This part of the experiment compares the classification accuracy and the number of features between the original data and the feature subset selected by the BONJE algorithm. The comparison results are shown in Table 3. The neighborhood radius selected for different data sets are listed in the last column. In addition, the feature subsets selected by the BONJE algorithm for different data sets are shown in Table 4. Please note that the boldface indicates the better value in the comparison data.

From the comparison of average classification accuracy in Table 3, it can be seen that the average classification accuracy of the BONJE algorithm on the Wine, WDBC, and Ionosphere data sets is slightly lower than the original data by 0.2%, 0.2%, and 0.8%, respectively. The accuracy loss caused by the BONJE algorithm is controlled within 1%, which shows that the BONJE algorithm maintains the classification accuracy of the original data. The average classification accuracy of the BONJE algorithm on the WPBC, Colon, SRBCT, DLBCL, Leukemia, and Lung data sets is higher than the original data by 1.5%, 4.8%, 3.7%, 7.4%, 7.4%, 2.5%, respectively, which indicates that the BONJE algorithm eliminates many redundant features and improves the classification accuracy of the data set. From the comparison of feature number in Table 3, it can be seen that BONJE algorithm can delete redundant features without reducing the classification accuracy, especially in high-dimensional data sets. In summary, the BONJE algorithm can effectively select the optimal feature subset, and the feature selection result can maintain or improve the classification ability of the data set.

### 4.5. The Performance of BONJE Algorithm on Low-Dimensional Data Sets

This part of the experiment compares the BONJE algorithm with four other advanced feature selection algorithms in the low-dimensional data set from the perspective of the number of selected features and the classification accuracy of KNN and SVM classifiers. The four advanced feature selection algorithms are: (1) Classic Rough Set Algorithm (RS) [1], (2) Neighborhood Rough Set Algorithm (NRS) [40], (3) Covering Decision Algorithm (CDA) [41], (4) Maximum Decision Neighborhood Rough Set Algorithm (MDNRS) [35]. Table 5, Table 6 and Table 7 show the experimental results of five different feature selection algorithms.

Comprehensive analyses of Table 5, Table 6 and Table 7 show that for the Wine data set, CDA algorithm selects the least number of features, but the KNN classification accuracy and SVM classification accuracy of CDA algorithm are far lower than BONJE algorithm by 23.4% and 31.8% respectively, which indicates that CDA algorithm loses features with important information in the selection process; For WDBC data set, although BONJE algorithm has more selected features than other algorithms, the classification accuracy of BONJE algorithm under the two classifiers is higher than that of other algorithms; For WPBC data set, NRS algorithm and the CDA algorithm choose the least number of features, but their classification accuracy under the two classifiers is lower than BONJE algorithm; For Ionosphere data set, the classification accuracy of BONJE algorithm is relatively high compared to other algorithms, and the number of features selected by BONJE algorithm is smaller than other algorithms; In general, the average number of selected features of BONJE algorithm is less, and BONJE algorithm has the highest average classification accuracy under the two classifiers, which shows that BONJE algorithm has stable reduction ability and can improve the classification accuracy of data set in low-dimensional data.

### 4.6. The Performance of BONJE Algorithm on High-Dimensional Data Sets

This part of the experiment compares the BONJE algorithm with four other advanced entropy-based feature selection algorithms from the perspective of different high-dimensional data sets. The four entropy-based feature selection algorithms are: (1) the mutual entropy-based attribute reduction algorithm (MEAR) [42], (2) the entropy gain-based gene selection algorithm (EGGS) [17], (3) the EGGS algorithm combined with the Fisher score (EGES-FS) [29], (4) feature selection algorithm with the Fisher score based on decision neighborhood entropy (FSDNE) [18]. Table 8, Table 9, Table 10, Table 11 and Table 12 show the experimental results of five different entropy-based feature selection algorithms.

As shown in Table 8, the KNN classification accuracy and C4.5 classification accuracy of the BONJE algorithm are better than other algorithms. Although the SVM classification accuracy of the BONJE algorithm is slightly lower than that of the first-ranked MEAR algorithm by 0.9%, the average classification accuracy of the BONJE algorithm is much higher than the second-ranked FSDNE algorithm by 3.5%. In general, the BONJE algorithm has excellent performance on the Colon data set.

Table 9 shows that the KNN classification accuracy and C4.5 classification accuracy of the BONJE algorithm are better than other algorithms. Although the SVM classification accuracy of the BONJE algorithm is lower than that of the first-ranked FSDNE algorithm by 1.5%, the average classification accuracy of the BONJE algorithm is much higher than the second-ranked FSDNE algorithm by 4.2%. Therefore, BONJE has stable classification performance on the SRBCT data set.

According to the experimental results in Table 10, it can be clearly seen that the KNN classification accuracy, SVM classification accuracy and C4.5 classification accuracy of the BONJE algorithm are better than other algorithms. Compared with the BONJE algorithm, the MEAR and EGGS-FS algorithms select fewer features, but the average classification accuracy of the MEAR and EGGS-FS algorithms is much lower than the BONJE algorithm. Therefore, the BONJE algorithm can delete many redundant features on the DLBCL data set without reducing the data classification ability.

According to the results in Table 11, although the KNN classification accuracy of the BONJE algorithm is lower than that of the FSDNE algorithm, the SVM classification accuracy and C4.5 classification accuracy of the BONJE algorithm are as high as 95.8% and 94.4%, respectively. The average classification accuracy of the BONJE algorithm is 1.5% higher than that of the second-ranked FSDNE algorithm. Therefore, the BONJE algorithm can effectively select feature subsets on the Leukemia data set and improve the classification ability of the data set.

It can be seen from Table 12 that the number of features selected by the BONJE algorithm is relatively high compared with other algorithms, but the BONJE algorithm has the highest average classification accuracy. Therefore, the BONJE algorithm can effectively reduce noise and improve classification accuracy on the Lung data set.

Based on the above experimental results and analyses, the BONJE algorithm can effectively select feature subsets under high-dimensional data, and the feature selection results can improve the classification ability of the data set.

### 4.7. Comparison of BONJE Algorithm and Multiple Dimensionality Reduction Algorithms

To further verify the reduction performance and classification ability of the BONJE algorithm, this part of the experiment compares the BONJE algorithm with other 10 reduction algorithms from the perspective of the number of selected features and SVM classification accuracy on 3 representative tumor data sets (Colon, Leukemia, Lung). The ten different dimensionality reduction methods are: (1) the neighborhood rough set-based reduction algorithm (NRS) [35], (2) feature selection algorithm with Fisher linear discriminant (FLD-NRS) [32], (3) the gene selection algorithm based on locally linear embedding (LLE-NRS) [43], (4) the Relief algorithm [44] combined with the NRS algorithm(Relief + NRS) [35], (5) the fuzzy back-ward feature algorithm (FBFE) [44], (6) the binary differential evolution algorithm (BDE) [2], (7) the sequential forward selection algorithm (SFS) [29], (8) the Spearman’s rank correlation coefficient algorithm (SC2) [36], (9) the mutual information maximization algorithm (MIM) [2], (10) feature selection algorithm with the Fisher score based on decision neighborhood entropy (FSDNE) [18]. Table 13 and Table 14 show the experimental results of 11 dimensionality reduction algorithms.

According to the results in Table 13 and Table 14, the SVM classification accuracy of the BONJE and LLE-NRS algorithms on the Colon dataset is the same and ranked second, but the number of features selected by the LLE-NRS algorithm is twice that of BONJE algorithm. The SVM classification accuracy of the BONJE algorithm on the Colon data set is lower than that of the FLD-NRS algorithm, but the SVM classification accuracy of the BONJE algorithm on the Leukemia and Lung data sets is much higher than that of the FLD-NRS algorithm by 13% and 10.5%, respectively, which shows that the classification performance of the BONJE algorithm is more stable. Although the BDE algorithm selects the least number of features on the Colon data set, its SVM classification accuracy is only 75%, which indicates that the BDE algorithm loses some important features in the process of selecting feature subsets. The SVM classification accuracy of the BONJE algorithm on the Leukemia data set is 0.1% lower than that of the first-ranked SFS algorithm, and the number of selected features the BONJE algorithm is only one more than the SFS algorithm, so these two algorithms have similar performance on the Leukemia data set. Compared with other algorithms, the number of features selected by the BONJE algorithm on the Lung data set is higher, but the SVM classification accuracy of the BONJE algorithm is the highest. In general, the BONJE algorithm is at a medium level compared to other algorithms in terms of the number of selected features, and has the highest average classification accuracy in terms of SVM classification accuracy, which is enough to show that BONJE algorithm has a stable dimension reduction performance, and can select features with important classification information in the data set.

### 4.8. Statistical Analyses

To systematically explore the statistical significance of algorithm classification results, this part of the experiment introduces the Friedman statistic test [45] and Nemenyi test [46].

The calculation formula of Friedman statistic test is as follows:

(25) $$\chi_{F}^{2}=\frac{12N}{M\left(M+1\right)}\sum_{i=1}^{M}R_{i}^{2}-3N\left(M+1\right)$$

(26) $$F_{F}=\frac{\left(N-1\right)\chi_{F}^{2}}{N\left(M-1\right)-\chi_{F}^{2}}$$

where *M* is the number of algorithms, *N* is the number of data sets, and $R_{i}$ represents the average ranking of the classification accuracy of the *i*-th algorithm on all data sets. $F_{F}$ is an F-distribution with M−1 and $\left(M-1\right)\left(N-1\right)$ degrees of freedom.

If the null hypothesis, all algorithms have the same performance, is rejected, it means that the performance of the algorithms is significantly different. Then, the Nemenyi test is used as a post-hoc test for algorithm comparison. If the average ranking difference between the algorithms is greater than the critical distance CD, it means that the algorithm with a high average ranking is better than the algorithm with a low average ranking.

The calculation formula of the critical distance CD is as follows:

(27) $$CD=q_{\alpha }\sqrt{\frac{M\left(M+1\right)}{6N}}$$

where $q_{\alpha }$ is the critical list value of the test, α represents the significance level of Bonferroni-Dunn.

According to the classification accuracy results of Table 6 and Table 7 on low-dimensional data sets, the rankings of the five feature selection algorithms under the KNN and SVM classifiers are shown in Table 15 and Table 16, respectively. Please note that the content in parentheses in all tables is the classification accuracy under the corresponding classifier

According to the algorithm rankings in Table 15 and Table 16, the two evaluation measurement values (Friedman statistics $\chi_{F}^{2}$ and Iman-Davenport test $F_{F}$) of the five feature selection algorithms under the KNN and SVM classifiers are shown in Table 17.

**Figure 2:**
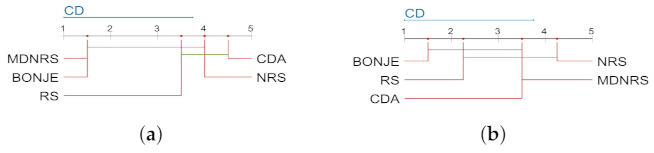
The five feature selection algorithms use the Nemenyi test under the two classifiers to compare the classification performance. (**a**) KNN. (**b**) SVM.

When the significance level α=0.1, the critical value of Friedman statistic test $F\left(4,12\right)=2.480$. It can be seen from Table 17 that the $F_{F}$ values under the KNN and SVM classifiers are both greater than $F\left(4,12\right)$, so the null hypothesis under the two classifiers is rejected. Then Nemenyi test is used as a post-hoc test to compare the algorithm performance, and the comparison results are shown in Figure 2. It is worth noting that the average ranking of each algorithm is plotted along the axis in the graph, and the best ranking in the axis is on the left. In particular, when there are thick lines between the algorithms, it means that the classification capabilities of these algorithms are similar, otherwise, they will be regarded as significantly different from each other [47].

It can be clearly seen from Figure 2 that BONJE algorithm ranks first under the two classifiers. The classification performance of the BONJE, MDNRS, RS and NRS algorithms under the KNN classifier is similar, and the BONJE algorithm is significantly better than the CDA algorithm. Under the SVM classifier, the classification performance of BONJE, RS, CDA and MDNRS algorithms is similar, and the BONJE algorithm performs better than the NRS algorithm

According to the classification accuracy results of Table 8, Table 9, Table 10, Table 11 and Table 12 on high-dimensional data sets, the rankings of the entropy-based feature selection algorithms under the KNN, C4.5 and SVM classifiers are shown in Table 18, Table 19 and Table 20, respectively.

According to the algorithm rankings in Table 18, Table 19 and Table 20, the two evaluation measurement values of the five entropy-based feature selection algorithms under the KNN, SVM, and C4.5 classifiers are shown in Table 21.

**Figure 3:**
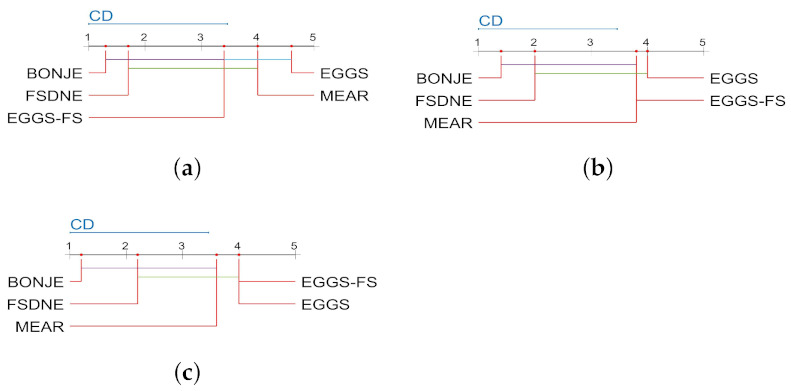
The five entropy-based feature selection algorithms use the Nemenyi test under the three classifiers to compare the classification performance. (**a**) KNN. (**b**) SVM. (**c**) C4.5.

When the significance level α=0.1, the critical value of Friedman statistic test $F\left(4,16\right)=2.333$, so null hypothesis under the three classifiers is rejected. The Nemenyi test is used as a post-hoc test to compare the performance of the algorithms, and the comparison results are shown in Figure 3.

According to the results in Figure 3, it can be seen that the ranking of BONJE algorithm is the best under the three classifiers. Under the KNN classifier, the classification performance of the BONJE, FSDNE and EGGS-FS algorithms is similar and the BONJE algorithm is significantly better than the MEAR and EGGS algorithms. Under the SVM classifier, the classification performance of the BONJE, FSDNE, EGGS-FS and FSDNE algorithms is similar, and the BONJE algorithm performs better than the EGGS algorithm. Under the C4.5 classifier, the BONJE algorithm has better classification performance than the EGGS and EGGS-FS algorithms.

According to the classification accuracy results of Table 14 on three representative tumor data sets, the rankings of the 11 dimensionality reduction algorithms under the SVM classifier are shown in Table 22.

**Figure 4:**
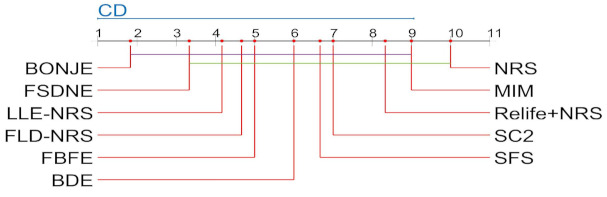
The 11 dimensionality reduction algorithms use the Nemenyi test under the SVM classifiers to compare the classification performance.

According to the ranking in Table 22, the $\chi_{F}^{2}=17.0491$ and $F_{F}=2.6329$ of the 11 dimensionality reduction algorithms under the SVM classifier. When the significance level α=0.1, the critical value of Friedman statistic test $F\left(10,20\right)=1.9367$. $F_{F}=2.8329$ is greater than $F\left(10,20\right)$, so the null hypothesis under the SVM classifier is rejected. The Nemenyi test is used as a post-hoc test to compare the algorithm performance, and the comparison result is shown in Figure 4.

Figure 4 shows that the dimensionality reduction effect of BONJE is significantly better than NRS algorithm. In addition, BONJE algorithm has the highest ranking, which shows that BONJE algorithm has stable classification performance compared to other algorithms.

In general, the classification results of BONJE algorithm under different data sets are significantly better than different algorithms, which shows that the classification performance of BONJE algorithm is more stable and efficient from a statistical point of view.

## 5. Conclusions

Since the classification performance of many feature selection algorithms based on rough set theory and its extension is not ideal, this paper proposes a feature selection algorithm combining information theory view and algebraic view in the neighborhood decision system to deal with redundant features and noise in data. First, some uncertainty measures of the neighborhood information entropy are studied to measure the uncertainty of knowledge in the neighborhood decision system. In addition, the credibility and coverage are introduced into the neighborhood decision system, and then neighborhood credibility and neighborhood coverage are defined and introduced into neighborhood joint entropy. Finally, based on the information theory view and algebraic view in the neighborhood decision system, a heuristic non-monotonic feature selection algorithm is proposed. A series of comparative experiments and statistical analysis results on four low-dimensional data sets and five high-dimensional data sets show that the algorithm can effectively remove redundant features and select the optimal feature subset. Since the BONJE algorithm needs to frequently calculate the neighborhood information particles of all samples, it has a high time complexity when processing high-dimensional data. Moreover, the BONJE algorithm cannot completely balance the classification level of the selected feature subset. In future work, it is necessary to study more effective search methods and uncertainty evaluation criteria to reduce the time complexity and classification error of the algorithm.

## Figures and Tables

**Table 1:** NDS.

*U*	*a*	*b*	*c*	*d*
$x_{1}$	0.12	0.41	0.61	Y
$x_{2}$	0.21	0.15	0.14	Y
$x_{3}$	0.31	0.11	0.26	N
$x_{4}$	0.61	0.13	0.23	N

**Table 2:** Description of the nine data sets.

No.	Data Sets	Features	Samples	Classes	Reference
1	Wine	13	178	3(59/71/48)	Fan et al. [35]
2	WDBC	30	569	2(357/ 212)	Fan et al. [35]
3	WPBC	32	194	2(46/148)	Fan et al. [35]
4	Ionosphere	34	351	2(126/225)	Fan et al. [35]
5	Colon	2000	62	2(22/40)	Xu et al. [36]
6	SRBCT	2308	63	4(23/8/12/20)	Tibshirani et al. [37]
7	DLBCL	5469	77	2(58/19)	Wang et al. [20]
8	Leukemia	7129	72	2(47/25)	Dong et al. [38]
9	Lung	12533	181	2(31/150)	Sun et al. [39]

**Table 3:** The classification results of the original data and the data processed by BONJE algorithm.

Data Sets	Raw Data					BONJE Algorithm					δ
	Features	KNN	SVM	C4.5	AVE	Features	KNN	SVM	C4.5	AVE	
Wine	13	0.949	0.983	0.938	**0.957**	**7**	0.961	0.961	0.944	0.955	0.1
WDBC	30	0.968	0.977	0.933	**0.959**	**7**	0.960	0.963	0.947	0.957	0.05
WPBC	32	0.701	0.763	0.758	0.741	**7**	0.743	0.763	0.763	**0.756**	0.1
Ionosphere	34	0.866	0.886	0.915	**0.889**	**13**	0.875	0.849	0.915	0.881	0.05
Colon	2000	0.758	0.855	0.823	0.812	**8**	0.840	0.840	0.903	**0.860**	0.25
SRBCT	2308	0.810	0.984	0.825	0.873	**5**	0.921	0.921	0.889	**0.910**	0.15
DLBCL	5469	0.909	0.974	0.727	0.870	**8**	0.948	0.948	0.935	0.**944**	0.3
Leukemia	7129	0.833	0.986	0.792	0.870	**8**	0.931	0.958	0.944	**0.944**	0.3
Lung	12533	0.939	0.994	0.950	0.961	**16**	0.994	0.994	0.967	**0.986**	0.45

**Table 4:** Feature subset selected on data set by BONFDE algorithm.

Data Sets	Feature Subset
Wine	{10,13,8,12,1,3,4}
WDBC	{11,22,10,29,25,21,27}
WPBC	{27,3,22,31,12,9,11}
Ionosphere	{21,11,4,29,30,5,16,34,26,27,20,19}
Colon	{1047,1672,29,354,1037,11,734,625}
SRBCT	{1954,2240,879,1716,1207}
DLBCL	{856,4656,1698,2651,3627,4410,3139,2618}
Leukemia	{758,2267,6041,1234,5503,6209,4184,2295}
Lung	{3916,5239,2193,3389,8110,8369,11272,2203,3466,610,
	12262,2139,1521,5858,3975,3334 }

**Table 5:** The number of selected features by the five feature selection algorithms on the low-dimensional data set.

Data Sets	RS	NRS	CDA	MDNRS	BONJE
Wine	5	3	**2**	4	7
WDBC	8	**2**	**2**	**2**	7
WPBC	7	**2**	**2**	4	7
Ionosphere	17	**8**	9	**8**	13
AVE	9.25	**3.75**	**3.75**	4.5	8.5

**Table 6:** KNN classification accuracy of five feature selection algorithms on low-dimensional data sets.

Data Sets	RS	NRS	CDA	MDNRS	BONJE
Wine	0.863	0.753	0.727	0.911	**0.961**
WDBC	0.911	0.923	0.923	0.930	**0.960**
WPBC	0.743	0.738	0.738	**0.761**	0.743
Ionosphere	0.866	0.859	0.848	**0.891**	0.875
AVE	0.846	0.818	0.809	0.873	**0.885**

**Table 7:** SVM classification accuracy of five feature selection algorithms on low-dimensional data sets.

Data Sets	RS	NRS	CDA	MDNRS	BONJE
Wine	0.640	0.402	0.643	0.910	**0.961**
WDBC	0.589	0.595	0.595	0.861	**0.963**
WPBC	**0.778**	0.757	0.757	0.692	0.763
Ionosphere	**0.881**	0.872	0.878	0.870	0.849
AVE	0.722	0.657	0.718	0.833	**0.884**

**Table 8:** Experimental results of five entropy-based feature selection algorithms on the Colon data set.

Algorithms	Features	KNN	SVM	C4.5	AVE
MEAR	5	0.770	**0.849**	0.822	0.814
EGGS	11	0.649	0.556	0.646	0.617
EGGS-FS	**2**	0.702	0.621	0.672	0.665
FSDNE	3	**0.840**	0.838	0.796	0.825
BONJE	8	**0.840**	0.840	**0.903**	**0.860**

**Table 9:** Experimental results of five entropy-based feature selection algorithms on the SRBCT data set.

Algorithms	Features	KNN	SVM	C4.5	AVE
MEAR	**1**	0.389	0.364	0.365	0.373
EGGS	12	0.575	0.703	0.513	0.597
EGGS-FS	**1**	0.637	0.651	0.626	0.638
FSDNE	9	0.846	**0.936**	0.821	0.868
BONJE	5	**0.921**	0.921	**0.889**	**0.910**

**Table 10:** Experimental results of five entropy-based feature selection algorithms on the DLBCL data set.

Algorithms	Features	KNN	SVM	C4.5	AVE
MEAR	**2**	0.765	0.777	0.778	0.773
EGGS	20	0.854	0.781	0.826	0.820
EGGS-FS	3	0.870	0.841	0.801	0.837
FSDNE	11	0.946	0.927	0.903	0.925
BONJE	8	**0.948**	**0.948**	**0.935**	**0.944**

**Table 11:** Experimental results of five entropy-based feature selection algorithms on the Leukemia data set.

Algorithms	Features	KNN	SVM	C4.5	AVE
MEAR	**3**	0.928	0.920	0.934	0.927
EGGS	8	0.629	0.802	0.733	0.721
EGGS-FS	5	0.801	0.680	0.813	0.765
FSDNE	9	**0.952**	0.929	0.905	0.929
BONJE	8	0.931	**0.958**	**0.944**	**0.944**

**Table 12:** Experimental results of five entropy-based feature selection algorithms on the Lung data set.

Algorithms	Features	KNN	SVM	C4.5	AVE
MEAR	**6**	0.958	0.929	0.964	0.950
EGGS	12	0.859	0.960	0.966	0.928
EGGS-FS	**6**	0.979	0.990	0.955	0.975
FSDNE	8	0.987	0.988	**0.979**	0.985
BONJE	16	**0.994**	**0.994**	0.967	**0.986**

**Table 13:** The number of features selected by 11 dimensionality reduction algorithms.

Algorithms	Colon	Leukemia	Lung	AVE
NRS	4	**5**	**3**	**4**
FLD-NRS	6	6	**3**	5
LLE-NRS	16	22	16	18
Relife+NRS	9	17	23	16.33
FBFE	35	30	80	48.33
BDE	**3**	7	**3**	4.33
SFS	19	7	**3**	9.67
SC2	4	**5**	**3**	**4**
MIM	19	7	**3**	9.67
FSDNE	**3**	9	8	6.67
BONJE	8	8	16	10.67

**Table 14:** SVM classification accuracy of 11 dimensionality reduction algorithms.

Algorithms	Colon	Leukemia	Lung	AVE
NRS	0.611	0.645	0.641	0.632
FLD-NRS	**0.880**	0.828	0.889	0.866
LLE-NRS	0.840	0.868	0.907	0.872
Relife+NRS	0.564	0.563	0.919	0.682
FBFE	0.833	0.912	0.852	0.866
BDE	0.750	0.824	0.980	0.851
SFS	0.521	**0.959**	0.833	0.771
SC2	0.805	0.852	0.806	0.821
MIM	0.653	0.727	0.795	0.725
FSDNE	0.828	0.928	0.988	0.915
BONJE	0.840	0.958	**0.994**	**0.931**

**Table 15:** Classification accuracy ranking of five feature selection algorithms under KNN classifier.

Data Sets	RS	NRS	CDA	MDNRS	BONJE
Wine	3(0.863)	4(0.753)	5(0.727)	2(0.911)	**1(0.961)**
WDBC	5(0.911)	3.5(0.923)	3.5(0.923)	2(0.930)	**1(0.960)**
WPBC	3(0.740)	4.5(0.738)	4.5(0.738)	**1(0.761)**	2(0.743)
Ionosphere	3(0.866)	4(0.859)	5(0.848)	**1(0.891)**	2(0.875)
Ave	3.5	4	4.5	**1.5**	**1.5**

**Table 16:** Classification accuracy ranking of five feature selection algorithms under SVM classifier.

Data Sets	RS	NRS	CDA	MDNRS	BONJE
Wine	4(0.640)	5(0.402)	3(0.643)	2(0.910)	**1(0.961)**
WDBC	3(0.598)	4.5(0.595)	4.5(0.595)	2(0.861)	**1(0.963)**
WPBC	**1(0.778)**	3.5(0.757)	3.5(0.757)	5(0.692)	2(0.763)
Ionosphere	**1(0.881)**	4(0.832)	3(0.848)	5(0.830)	2(0.849)
Ave	2.25	4.25	3.5	3.5	**1.5**

**Table 17:** $\chi_{F}^{2}$ and $F_{F}$ under two classifiers of five feature selection algorithms.

	KNN	SVM
$\chi_{F}^{2}$	12.8	7.8
$F_{F}$	12	2.8537

**Table 18:** Classification accuracy ranking of five entropy-based feature selection algorithms under KNN classifier.

Data Sets	MEAR	EGGS	EGGS-FS	FSDNE	BONJE
Colon	3(0.770)	5(0.649)	4(0.702)	**1.5(0.840)**	**1.5(0.840)**
SRBCT	5(0.389)	4(0.575)	3(0.637)	2(0.846)	**1(0.921)**
DLBCL	5(0.765)	4(0.854)	3(0.870)	2(0.946)	**1(0.948)**
Leukemia	3(0.928)	5(0.629)	4(0.901)	**1(0.952)**	2(0.931)
Lung	4(0.958)	5(0.859)	3(0.979)	2(0.987)	**1(0.994)**
AVE	4	4.6	3.4	1.7	**1.3**

**Table 19:** Classification accuracy ranking of five entropy-based feature selection algorithms under SVM classifier.

Data Sets	MEAR	EGGS	EGGS-FS	FSDNE	BONJE
Colon	**1(0.849)**	5(0.556)	4(0.621)	3(0.838)	2(0.840)
SRBCT	5(0.364)	3(0.703)	4(0.651)	**1(0.936)**	2(0.921)
DLBCL	5(0.777)	4(0.781)	3(0.841)	2(0.927)	**1(0.948)**
Leukemia	3(0.920)	4(0.802)	5(0.680)	2(0.929)	**1(0.958)**
Lung	5(0.929)	4(0.960)	3(0.990)	2(0.988)	**1(0.994)**
AVE	3.8	4	3.8	2	**1.4**

**Table 20:** Classification accuracy ranking of five entropy-based feature selection algorithms under C4.5 classifier.

Data Sets	MEAR	EGGS	EGGS-FS	FSDNE	BONJE
Colon	2(0.822)	5(0.646)	4(0.672)	3(0.796)	**1(0.903)**
SRBCT	5(0.365)	4(0.513)	3(0.626)	2(0.821)	**1(0.889)**
DLBCL	5(0.778)	3(0.826)	4(0.801)	2(0.903)	**1(0.935)**
Leukemia	2(0.934)	5(0.733)	4(0.813)	3(0.905)	**1(0.944)**
Lung	4(0.964)	3(0.966)	5(0.955)	**1(0.979)**	2(0.967)
AVE	3.6	4	4	2.2	**1.2**

**Table 21:** $\chi_{F}^{2}$ and $F_{F}$ under three classifiers of five entropy-based feature selection algorithms.

	KNN	SVM	C4.5
$\chi_{F}^{2}$	16.6	11.68	12.48
$F_{F}$	19.5294	5.6154	6.6383

**Table 22:** Classification accuracy ranking of eleven dimensionality reduction algorithms under SVM classifier.

Algorithms	Colon	Leukemia	Lung	AVE
NRS	9(0.611)	10(0.645)	11(0.641)	10
FLD-NRS	**1(0.880)**	7(0.828)	6(0.889)	4.67
LLE-NRS	2.5(0.840)	5(0.868)	5(0.907)	4.17
Relife+NRS	10(0.564)	11(0.563)	4(0.919)	8.33
FBFE	4(0.833)	4(0.912)	7(0.852)	5
BDE	7(0.750)	8(0.824)	3(0.980)	6
SFS	11(0.521)	**1(0.959)**	8(0.833)	6.67
SC2	6(0.805)	6(0.852)	9(0.806)	7
MIM	8(0.653)	9(0.727)	10(0.795)	9
FSDNE	5(0.828)	3(0.928)	2(0.988)	3.33
BONJE	2.5(0.840)	2(0.958)	**1(0.994)**	**1.83**

## Data Availability

Not applicable.

## References

[1] Pawlak Z. (2002). Rough sets and intelligent data analysis. Inf. Sci..

[2] Sun L., Zhang X.Y., Xu J.C., Zhang S.G. (2019). An Attribute Reduction Method Using Neighborhood Entropy Measures in Neighborhood Rough Sets. Entropy.

[3] Zhao R.Y., Zhang H., Li C.L. (2005). Research on Discretization Model of Continuous Attributes of Rough Sets and Analysis of Main Points of Application. Comput. Eng. Appl..

[4] Shu W.H., Qian W.B. (2020). Incremental feature selection for dynamic hybrid data using neighborhood rough set. Knowl. Based Syst..

[5] Sun L., Wang L.Y. (2020). Neighborhood multi-granulation rough sets-based attribute reduction using Lebesgue and entropy measures in incomplete neighborhood decision systems. Knowl. Based Syst..

[6] Wang C.Z., Huang Y. (2020). Feature Selection Based on Neighborhood Self-Information. IEEE Trans. Cybern..

[7] Miao D.Q. (2001). Discretization of continuous attributes in rough set theory. Acta Autom. Sin..

[8] Wang C.Z., Shi Y.P., Fan X.D., Shao M.W. (2019). Attribute reduction based on k-nearest neighborhood rough sets. Int. J. Approx. Reason..

[9] Chen Y.M., Qin N., Li W., Xu F.F. (2019). Granule structures, distances and measures in neighborhood systems. Knowl. Based Syst..

[10] Yao Y.Y. (1998). Relational interpretations of neighborhood operators and rough set approximation opera-tors. Inf. Sci..

[11] Hu Q.H., Yu D.R., Liu J.F. (2008). Neighborhood rough set based heterogeneous feature subset selection. Inf. Sci..

[12] Sun L., Wang W., Xu J.C., Zhang S.G. (2019). Improved LLE and neighborhood rough sets-based gene selection using Lebesgue measure for cancer classification on gene expression data. J. Intell. Fuzzy Syst..

[13] Sahlol A.T., Kim S. (2020). Handwritten Arabic Optical Character Recognition Approach Based on Hybrid Whale Optimization Algorithm With Neighborhood Rough Set. IEEE Access.

[14] Feng L., Li C., Chen L. (2013). Facial expression feature selection method based on neighborhood rough set and quantum genetic algorithm. J. Hefei Univ. Technol..

[15] Wong S.K.M., Ziarko W. (1985). On optimal decision rules in decision tables. Bull. Pol. Acad. Sci. Math..

[16] Jiang Z.H., Liu K.Y., Yang X.B., Yu H.L., Fujitac H., Qian Y.H. (2020). Accelerator for supervised neighborhood based attribute reduction. Int. J. Approx. Reason..

[17] Chen Y.M., Zhang Z.J., Zheng J.Z., Ma Y., Xue Y. (2017). Gene selection for tumor classification using neighborhood rough sets and entropy measures. J. Biomed. Inform..

[18] Sun L., Zhang X.Y., Qian Y.H., Xu J.C., Zhang S.G. (2019). Feature selection using neighborhood entropy-based uncertainty measures for gene expression data classification. Inf. Sci..

[19] Li J.T., Dong W.P., Meng D.Y. (2018). Grouped gene selection of cancer via adaptive sparse group lasso based on conditional mutual information. IEEE-ACM Trans. Comput. Biol. Bioinform..

[20] Wang C.Z., Hu Q.H., Wang X.Z., Chen D.G., Qian Y.H., Dong Z. (2018). Feature selection based on neighborhood discrimination index. IEEE Trans. Neural Netw. Learn. Syst..

[21] Wang C.Z., Huang Y. (2021). Attribute reduction with fuzzy rough self-information measures. Inf. Sci..

[22] Tsumoto S. (2002). Accuracy and coverage in rough set rule induction. Proceedings of the International Conference on Rough Sets and Current Trends in Computing.

[23] Xu J.C., Wang Y. (2019). Feature genes selection based on fuzzy neighborhood conditional entropy. J. Intell. Fuzzy Syst..

[24] Sun L., Wang L.Y. (2021). Feature Selection Using Fuzzy Neighborhood Entropy-Based Uncertainty Measures for Fuzzy Neighborhood Multigranulation Rough Sets. IEEE Trans. Fuzzy Syst..

[25] Sun L., Yin T.Y. (2020). Multilabel feature selection using ML-ReliefF and neighborhood mutual information for multilabel neighborhood decision systems. Inf. Sci..

[26] Sun L., Wang L.Y. (2019). Feature selection using Lebesgue and entropy measures for incomplete neighborhood decision systems. Knowl. Based Syst..

[27] Wang L., Ye J. (2013). Matrix method of knowledge granularity calculation and its application in attribute reduction. Comput. Eng. Sci..

[28] Wang L., Li T.R. (2013). A method of knowledge granularity calculation based on matrix. Pattern Recognit. Artif. Intell..

[29] Sun L., Zhang X.Y. (2019). Joint neighborhood entropy-based gene selection method with fisher score for tumor classification. Appl. Intell..

[30] Miao D.Q., Hu G.R. (1999). A heuristic algorithm for knowledge reduction. J. Comput. Res. Dev..

[31] Wang G.Y., Yang D.C. (2002). Decision table reduction based on conditional information entropy. Chin. J. Comput..

[32] Sun L., Zhang X.Y., Xu J.C., Wang W., Liu R.N. (2018). A gene selection approach based on the fisher linear discriminant and the neighborhood rough set. Bioengineered.

[33] Aziz R., Verma C.K., Srivastava N. (2016). A fuzzy based feature selection from independent component subspace for machine learning classification of microarray data. Genom. Data.

[34] Jiang F., Sui Y.F., Zhou L. (2015). A relative decision entropy-based feature selection approach. Pattern Recognit..

[35] Fan X.D., Zhao W.D., Wang C.Z., Huang Y. (2018). Attribute reduction based on max-decision neighborhood rough set model. Knowl. Based Syst..

[36] Xu J.C., Mu H.Y., Wang Y., Huang F.Z. (2018). Feature genes selection using supervised locally linear embedding and correlation coefficient for microarray classification. Comput. Math. Med..

[37] Tibshirani R., Hastie T., Narasimhan B., Chu G. (2002). Diagnosis of multiple cancer types by shrunken centroids of gene expression. Proc. Natl. Acad. Sci. USA.

[38] Dong H.B., Li T., Ding R., Sun J. (2018). A novel hybrid genetic algorithm with granular information for feature selection and optimization. Appl. Soft Comput..

[39] Sun S.Q., Peng Q.K., Zhang X.K. (2016). Global feature selection from microarray data using Lagrange multipliers. Knowl. Based Syst..

[40] Yang X.B., Zhang M., Dou H.L., Yang J.Y. (2011). Neighborhood systems-based rough sets in incomplete information system. Knowl. Based Syst..

[41] Yang J., Liu Y.L., Feng C.S., Zhu G.Q. (2016). Applying the Fisher score to identify Alzheimer’s disease-related genes. Genet. Mol. Res..

[42] Xu F.F., Miao D.Q., Wei L. (2009). Fuzzy-rough attribute reduction via mutual information with an application to cancer classification. Comput. Math. Appl..

[43] Sun L., Xu J.C., Wang W., Yin Y. (2016). Locally linear embedding and neighborhood rough set-based gene selection for gene expression data classification. Genet. Mol. Res..

[44] Zhang W., Chen J.J. (2018). Relief feature selection and parameter optimization for support vector machine based on mixed kernel function. J. Mater. Eng. Perform..

[45] Dunn Q.J. (1961). Multiple comparisons among means. J. Am. Stat. Assoc..

[46] Friedman M. (1940). A comparison of alternative tests of significance for the problem of mrankings. Ann. Math. Stat..

[47] Lin Y.J., Li Y.W., Wang C.X., Chen J.K. (2018). Attribute reduction for multi-label learning with fuzzy rough set. Knowl. Based Syst..

